# Analysis of physiological signals for recognition of boredom, pain, and surprise emotions

**DOI:** 10.1186/s40101-015-0063-5

**Published:** 2015-06-18

**Authors:** Eun-Hye Jang, Byoung-Jun Park, Mi-Sook Park, Sang-Hyeob Kim, Jin-Hun Sohn

**Affiliations:** IT Convergence Technology Research Laboratory, Electronics Telecommunication Research Institute, 218 Gajeong-ro, Yuseong-gu, Daejeon 305-705 South Korea; Department of Psychology, Chungnam National University, 99 Daehak-ro, Yuseong-gu, Daejeon 305-764 South Korea

**Keywords:** Emotion, Physiological signal, Emotion recognition

## Abstract

**Background:**

The aim of the study was to examine the differences of boredom, pain, and surprise. In addition to that, it was conducted to propose approaches for emotion recognition based on physiological signals.

**Methods:**

Three emotions, boredom, pain, and surprise, are induced through the presentation of emotional stimuli and electrocardiography (ECG), electrodermal activity (EDA), skin temperature (SKT), and photoplethysmography (PPG) as physiological signals are measured to collect a dataset from 217 participants when experiencing the emotions. Twenty-seven physiological features are extracted from the signals to classify the three emotions. The discriminant function analysis (DFA) as a statistical method, and five machine learning algorithms (linear discriminant analysis (LDA), classification and regression trees (CART), self-organizing map (SOM), Naïve Bayes algorithm, and support vector machine (SVM)) are used for classifying the emotions.

**Results:**

The result shows that the difference of physiological responses among emotions is significant in heart rate (HR), skin conductance level (SCL), skin conductance response (SCR), mean skin temperature (meanSKT), blood volume pulse (BVP), and pulse transit time (PTT), and the highest recognition accuracy of 84.7 % is obtained by using DFA.

**Conclusions:**

This study demonstrates the differences of boredom, pain, and surprise and the best emotion recognizer for the classification of the three emotions by using physiological signals.

## Background

Emotions are known as multi-componential responses that are composed of coordinated changes in subjective feeling, motor expression, and physiological activation [1]. Additionally, they are processes directed towards a specific internal or external event or object, which result in changes in both behavior and bodily state (i.e., physiological change) [2, 3]. Emotions increase our chances of survival by providing us with the ability to deal with sudden events in our surroundings [4]. In a positive state, optimistic feelings dominate and cognitive functions (e.g., problem-solving abilities) are improved. On the other hand, in negative states, pessimistic feelings dominate, our capacities are underestimated and analytical thinking is increased [5, 6]. In particular, because emotion plays an important role in contextual understanding of messages from others in speech or visual forms (i.e., facial expressions, body gestures), it has been recognized as one of the most important ways of people to communicate with each other.

Recently, many attempts have been made in human–human or human–computer interaction (HCI) for robots or machines to improve their abilities to understand humans’ intentions or affective states. The accurate emotion recognition would allow computers to understand humans’ emotions and to interact with humans in accordance with humans’ affective states for better communications and more natural interactions between human and computers [7]. For effective understanding of emotions, emotion recognition systems based on physiological signals have been demonstrated by Picard and colleagues at the MIT Media Laboratory [8] and other previous researchers [9] resulting in the recognition accuracy of more than 80 % on average. Physiological signals have some advantages as the following although they may be venerable to artifact caused by motions or other external factors. First, the acquisition of physiological signals by noninvasive sensors is relatively simple and it makes us possible to monitor users’ autonomic activity associated with emotional or cognitive states in real time. Second, physiological responses are robust to social masking or factitious emotion expressions since they can be acquired through spontaneous emotional responses and are less sensitive in social and cultural difference [10]. For those reasons, in the field of psychophysiology, the investigation of human emotional status has been based on the analysis of physiological signals from both the central and autonomic nervous systems [11, 12].

Affective computing by using psychophysiology, basic emotions such as happiness, anger, sadness, fear, disgust, and surprise have been studied commonly. For example, Kreibig [13] has examined the relationship between basic emotions and physiological responses suggesting typical response pattern of each emotion based on the review of 134 articles. Although she has investigated emotion-specific physiological responses among basic emotions, she failed to identify the surprise-specific response due to the limited number of studies on the emotion. The surprise among the basic emotions has been rarely investigated. Additionally, the relationship between non-basic emotions and physiological signals has not been revealed yet. Since humans’ emotions experienced in their daily lives are very delicate and complex and in order to understand the human emotions better, non-basic and social emotions are needed to be studied. For this purpose, it is needed to understand the physiological underpinning, underlying basic emotions, particularly, surprise and non-basic emotions.

Therefore, we have focused the relationship between basic emotions, particularly, surprise and non-basic emotions, i.e., boredom and pain and physiological responses. For this purpose, firstly, we were to identify the difference of physiological responses for the three emotions. Secondly, we aimed to classify the three emotions by using discriminant function analysis (DFA) as a statistical method and machine learning algorithms to test the possibility whether the three emotions could be classified and applied in the field of affective computing. The machine learning algorithms used in the study were five preferred emotion recognizers, i.e., linear discriminant analysis (LDA), classification and regression trees (CART), self-organizing map (SOM), Naïve Bayes algorithm, and support vector machine (SVM). The reason why we used six emotion recognizers is that we were to find the best classifier for boredom, pain, and surprise through the comparative analysis of the results of classification based on the physiological signals. Before the explanation of the experimental methods, we included the operational definitions of the three emotions.

### The definition of emotions: boredom, pain, and surprise

Boredom is an emotional experience when an individual is left without anything in particular to do and not interested in his/her surroundings. It has been defined as an unpleasant, transient affective state in which the individual feels a pervasive lack of interest and difficulty concentrating on the current activity. Leary and colleagues [14] describe boredom as an affective experience associated with cognitive attention processes. In positive psychology, boredom is defined as a response to a moderate challenge for which the human has more than enough skill [15].

Regarding definition of pain, it is an unpleasant sensory and emotional experience associated with actual or potential tissue damage, or described in terms of such damage according to the International Association for the Study of Pain. It is divided into physical pain and psychological pain. The former is a feeling of the nerves telling the brain that there is a physical sensation causing discomfort in the present. In this study, although physical pain may be possible to have direct physiological responses to the physical stimulus originated from autonomic nervous system, without much relevance with “emotional response” from the brain, we have used a stimulus to induce physical pain. It could be attributed from various causes when psychological needs such as the need for love, autonomy, affiliation, and achievement are frustrated, or the need to avoid harm, shame, and embarrassment occurs [16]. It is likely to be evoked mixing with other emotions. For example, Vangelisti [17] describes psychological pain as a blend of fear and sadness. It could be accompanied by other emotions, including fear, sadness, anger, anxiety, and shame [18–20]. Since the psychological pain is so complex due to the difficulty of defining and provoking, we have chosen physical pain in the study.

Surprise is defined as a transient emotional state experienced resulting from an unexpected event and can have any intensity and valence, i.e., neutral/moderate and pleasant/unpleasant, respectively [21]. It can be divided into “wonder” that people feel when perceiving something rare or unexpected [22] and “startle” response that is generated by a sudden stimulus such as a flash of light, a loud noise, or a quick movement [23, 24]. Considering that the surprise experienced induces the startle response commonly and the main function of startle response is to interrupt an ongoing action and reorient attention to a new and possibly significant event, we have included surprise emotion as a startle response with negative valence in this study.

## Experimental methods

### Emotional stimuli

The selected emotional stimuli are shown in Table 1. The stimulus for boring induction was the combination of a presentation of “+” symbol on screen and a repetitive sound of numbers from 1 to 10 for 3 min. For provoking pain, standard blood pressure with a maximum pressure of 300 mmHg was given while the participant is wearing a blood pressure cuff on his/her non-dominant arm for 1 min. The surprise-provoking stimulus was the sudden presentation of the sounds of the hog-caller, breaking glass, and thunder while the participants concentrated on a game-like computer task for 1 min.

**Table 1:** Examples of emotion stimuli

Emotion	Stimulus
Boredom	
	Repetitive sounds of numbers from 1 to 10 (3 min)
Pain	
	Induction of pain by using a blood pressure cuff (1 min)
Surprise	
	Sudden sounds of hog-caller, breaking glass, and thunder during concentration on a game-like computer task (1 min)

These stimuli have been verified for their appropriateness and effectiveness through a preliminary psychometric experiment. One hundred and twenty-two college students participated in the experiment. They rated their experienced emotions during exposure to each emotional stimulus. In the experiment, the appropriateness of emotional stimulus means a consistency between the intended emotion by an experimenter and feeling experienced by the participants. It can be demonstrated as (the number of participants who reported the intended emotion/the number of total participants) *100 by using mathematical expressions. The effectiveness is an intensity of emotions that participants rated on a 1-to-7-point Likert-type scale (e.g., 1 being “least weak” and 7 being “most intense”). The averages (SD) of appropriateness and effectiveness for these stimuli are as follows: the stimulus inducing boredom had 86.0 % and 5.23(1.36), pain inducing stimulus was 97.3 % and 4.96(1.34), and 94.1 % and 6.12(1.14) in surprise.

### Experimental procedure

Two hundred seventeen healthy (97 males and 120 females) aged 20.0 (SD 1.80) years old college students participated in this experiment. They had no history of medical illness attributed to heart disease, respiration, or central nervous system disorder. They were introduced to the experiment protocols and filled out a written consent before the beginning of experiment. Also, they were paid $30 USD per session to compensate for their participation. Prior to the experiment, they were introduced to experiment procedures in details and have an adaptation time to feel comfortable in the laboratory setting. Then, electrodes are attached on their wrist, finger, and ankle for measurement of physiological signals. Physiological signals are measured for 1 min during relaxation while viewing the “fixation” on the computer screen (baseline state) and for 1~3 min during the presentation of emotional stimuli (emotional state), and then an additional 1 min after presentation of the emotional stimuli (recovery state). After the physiological signal acquisition, psychological assessment was conducted. During the assessment, participants were asked to label the experienced emotion (i.e., happiness, boredom, sadness, fear, anger, disgust, surprise, and others) and rate the intensity of the emotion in response to the emotional stimulus. The experimental procedure and the study protocol was approved by the Institutional Review Board of the Chungnam National University (No. 201309-SB-004-01).

### Physiological signals acquisition and feature extraction

The data of physiological signals were acquired by using the MP150 and AcqKnowledge v 4.1 (Biopac, USA). The signals were recorded at a 250-Hz sampling rate and were digitized by an analog-to-digital converter. Additionally, appropriate amplification and band-pass filtering were performed. The acquired signals were as follows.

Electrocardiography (ECG) is possible to gain an insight into the relative effects of the parasympathetic and sympathetic components at the nodes using a noninvasive recording technique. It is used to measure the rate and regularity of heartbeats, as well as the size and position of the chambers, the presence of any damage to the heart, and the effects of drugs or devices used to regulate the heart, such as a pacemaker. For acquisition of ECG signal, ECG electrodes (Meditrace 100, Kendall_LTP, USA) were placed on both wrists and one left ankle with two kinds of electrodes, sputtered and AgCl ones. The electrode on the left ankle was used as a reference.

Electrodermal activity (EDA) is one of physiological signals that can easily be measured from the body surface and represents the activity of the autonomic nervous system. It characterizes changes in the electrical properties of the skin due to the activity of sweat glands and is physically interpreted as conductance. Sweat glands distributed on the skin receive input from the sympathetic nervous system only, and thus this is a good indicator of arousal level due to external sensory and cognitive stimuli. EDA signal was measured with the use of 8-mm AgCl electrodes (TSD203, Biopac, USA) placed on the volar surface of the distal phalanges of the index and middle fingers of the non-dominant hand. The electrodes were filled with a 0.05 molar isotonic NaCl paste to provide a continuous connection between the electrodes and the skin.

Skin temperature (SKT) measures the thermal response of human skin. Variations in the SKT mainly come from localized changes in blood flow caused by vascular resistance or arterial blood pressure. Local vascular resistance is modulated by smooth muscle tone, which is mediated by the sympathetic nervous system. The mechanism of arterial blood pressure variation can be described by a complicated model of cardiovascular regulation by the autonomic nervous system. Thus, it is evident that the SKT variation reflects autonomic nervous system activity and is another effective indicator of emotional status. The SKT was measured from the fingertip. SKT signals were measured on the first joint of the non-dominant ring finger using a SKT100B amplifier and a fast response thermistor (TSD202A). SKT were calculated for each time unit from the raw SKT signals.

Photoplethysmography (PPG) is a process of applying a light source and measuring the light reflected by the skin. At each contraction of the heart, blood is forced through the peripheral vessels, producing engorgement of the vessels under the light source, thereby modifying the amount of light to the photo-sensor. PPG is a wave form signal that indicates pulsation of the chest wall and great arteries followed by a heartbeat, that is, the blood pressure and vascular diameter change with cardiac cycle, and it means that these arterial pulsatile alterations are propagating to the peripheral vascular system. It aims to observe on mechanical movement of heart and kinetics of blood flow and manifests the pulsation of the chest wall and great arteries followed by a heartbeat as a wave form. For recording of PPG, the sensor (TSD200, Biopac, USA) was attached on the first joint of the non-dominant thumb. The signals were amplified by each amplifiers, ECG100C, GSR100C, SKT100C, and PPG100C (Biopac, USA).

To extract features, the acquired signals were analyzed for 30 s each from the baseline state and the emotional state by using AcqKnowledge v 4.1 (Biopac, USA). Twenty-seven features were extracted from the obtained physiological signals (Table 2). In ECG, heart rate (HR), low-frequency heart rate variability spectral power [0.04~0.15 Hz] (LF), high-frequency heart rate variability spectral power [0.15~0.4 Hz] (HF), and ratio of low- to high-frequency power (LF/HF HRV) were extracted. The skin conductance level (SCL), average of skin conductance response (SCR) and number of skin conductance response are obtained from EDA. The mean SKT were calculated by averaging the SKT amplitude values during the 30-s baseline and emotional state each. The blood volume pulse (BVP) and pulse transit time (PTT) from PPG were also extracted. Five hundred and thirty-seven datasets of physiological signals were selected for data analysis after excluding the dataset due to severe artifact effects by movements, noises, etc.

**Table 2:** Extracted physiological features

Signals	Features
ECG	b_HR, b_LF, b_HF, b_HRV, e_HR, e_LF, e_HF, e_HRV, d_HR, d_LF, d_HF, d_HRV
EDA	b_SCL, b_SCR, e_SCL, e_SCR, d_SCL, d_SCR
SKT	b_meanSKT, e_meanSKT, d_meanSKT
PPG	b_BVP, b_PTT, e_BVP, e_PTT, d_BVP, d_PTT

*b_* baseline, *e_* emotional state, *d_* “e_” − “b_”, *ECG* electrocardiography, *EDA* electrodermal activity, *SKT* skin temperature, *PPG* photoplethysmography, *HR* heart rate, *LF* low-frequency, *HRV* heart rate variability, *SCL* skin conductance level, *SCR* skin conductance response, *BVP* blood volume pulse, *PTT* pulse transit time

## Emotion recognition methods

In order for the comparative analysis of emotion recognition, we use one statistical method and five machine learning algorithms. The six methods have been briefly described in this section. Refer to [25–31] for detail of the methods.

### Discriminant function analysis

Discriminant function analysis (DFA) is a statistical analysis used to predict a categorical dependent variable (called a grouping variable) by one or more continuous or binary independent variables (called predictor variables) [25]. The model is composed of a discriminant function based on linear combinations of independent variables, and those independent variables provide the best discrimination between groups. DFA is used to maximally separate the groups, to determine the most parsimonious way to separate groups, or to discard variables which are little related to group distinctions. DFA is similar to regression analysis. A discriminant score can be calculated based on the weighted combination of the independent variables.

1 $$ {D}_i=a+{b}_1{\mathrm{x}}_1+{b}_2{\mathrm{x}}_2+\dots +{b}_{\mathrm{n}}{\mathrm{x}}_{\mathrm{n}} $$

*D*_*i*_ is the predicted score (discriminant score), *x* is the predictor, and *b* is the discriminant coefficient.

When interpreting multiple discriminant functions, which arise from analyses with more than two groups and more than one continuous variable, the different functions are first tested for statistical significance. If the functions are statistically significant, then the groups can be distinguished based on predictor variables. The basic idea underlying discriminant function analysis is to determine whether groups differ with regard to the mean of a variable, and then to use that variable to predict group membership.

### Linear discriminant analysis

Linear discriminant analysis (LDA) which is one of the linear models is a method used in statistics, pattern recognition, and machine learning to find a linear combination of features which characterizes or separates two or more classes of objects or events. LDA finds the direction to project data on so that between-class variance in maximized and within-class variance in minimized, and then offers a linear transformation of predictor variables which provides a more accurate discrimination [26]. In LDA, the measurement space is transformed so that the separability between the emotional states is maximized. The separability between the emotional states can be expressed by several criteria.

LDA finds the direction to project data on so that between-class variance (*S*_*B*_) in maximized and within-class variance (*S*_*W*_) in minimized, and then offers a linear transformation of predictor variables which provides a more accurate discrimination. *S*_*W*_ is proportional to the sample covariance matrix for the pooled d-dimensional data. It is symmetric and positive semi-definite, and it is usually nonsingular if *n* > *d*. Likewise, *S*_*B*_ is also symmetric and positive semi-definite, but because it is the outer product of two vectors, its rank is at most one [26].

In terms of *S*_*B*_ and *S*_*W*_, the criterion function *J* is written as

2 $$ J\left(\mathrm{w}\right)=\left({\mathrm{w}}^T{S}_B\mathrm{w}\right)/\left({\mathrm{w}}^T{S}_W\mathrm{w}\right) $$

This expression is well known in mathematical physics and the generalized Rayleigh quotient. It is easy to show that a vector w that maximizes *J* must satisfy

3 $$ {S}_B\mathrm{w}=\uplambda {S}_W\mathrm{w} $$

For some constant λ, which is a generalized eigenvalue problem.

LDA works when the measurements made on independent variables for each observation are continuous quantities. When dealing with categorical independent variables, the equivalent technique is discriminant correspondence analysis.

### Classification and regression trees

Classification and regression tree (CART) [26, 27] is one of decision tree and nonparametric techniques that can select from among a large number of variables the most important ones in determining the outcome variable, given the data represented at a node, either declare that node to be a leaf (and state what category to assign to it), or find another property to use to split the data into subsets. This is a generic tree-growing methodology known as CART. The fundamental principle underlying tree creation is that of simplicity. We prefer decisions that lead to a simple, compact tree with few nodes. In formalizing this notion, the most popular measure is the entropy impurity (or occasionally information impurity):

4 $$ i(N)=-{\displaystyle \sum_jP\left({\omega}_j\right){ \log}_2P\left({\omega}_j\right)} $$

Where, *P*(*ω*_*j*_) is the fraction of patterns at node *N* that are in class *ω*_*j*_. By the well-known properties of entropy, if all the patterns are of the same category, the impurity is 0; otherwise, it is positive, with the greatest value occurring when the different classes are equally likely.

In most general terms, the purpose of the analyses via tree-building of CART is to determine a set of if-then logical (split) conditions that permit accurate prediction or classification of cases. It is relatively simple for non-statisticians to interpret. Decision rules based on trees are more likely to be feasible and practical, since the structure of the rule and its inherent logic are apparent.

### Self-organizing map

Self-organizing map (SOM) [26, 28], called Kohonen map, is a type of artificial neural networks in the unsupervised learning category and generally present a simplified, relational view of a highly complex dataset. This is called a topology-preserving map because there is a topological structure imposed on the nodes in the network. A topological map is simply a mapping that preserves neighborhood relations. The goal of training is that the “winning” unit in the target space is adjusted so that it is more like the particular pattern. Others in the neighborhood of output are also adjusted so that their weights more nearly match those of the input pattern. In this way, neighboring points in the input space lead to neighboring points being active. Given the winning unit *i*, the weight update is

5 $$ {\mathrm{w}}_i\left(\mathrm{new}\right)={\mathrm{w}}_i+{h}_{ci}\left(\mathrm{x}-{\mathrm{w}}_i\right) $$

6 $$ {h}_{ci}={h}_0\kern0.5em  \exp \left(-{\left\Vert {r}_i-{r}_c\right\Vert}^2/{\upsigma}^2\right) $$

where, *h*_*ci*_ is called the neighborhood function that has value 1 for *i* = *c* and smaller for large value of the distance between units *i* and *c* in the output array. *h*_0_ and σ are suitable decreasing functions of time. Units close to the winner as well as the winner itself have their weights updated appreciably. Weights associated with far away output nodes do not change significantly. It is here that the topological information is supplied.

### The Naïve Bayes algorithm

The Naïve Bayes algorithm [26] is a simple probabilistic classification algorithm based on applying Bayes’ rule with strong (naive) independent assumptions and particularly suited when the dimensionality of the inputs is high. Naïve Bayes classifier assumes that the presence (or absence) of a particular feature of a class is unrelated to the presence (or absence) of any other feature, given the class variable. This helps alleviate problems stemming from the curse of dimensionality, such as the need for datasets that scale exponentially with the number of features. While Naïve Bayes often fails to produce a good estimate for the correct class probabilities, this may not be a requirement for many applications.

When the dependency relationships among the features used by a classifier are unknown, we generally proceed by taking the simplest assumption, namely, that the feature are conditionally independent given the category, that is,

7 $$ p\left({\omega}_k\left|\mathbf{x}\right.\right)\propto {\displaystyle \prod_{i=1}^dp\left({x}_i\left|{\omega}_k\right.\right)} $$

This so-called Naïve Bayes rule often works quite well in practice, and it can be expressed by a very simple belief net.

### Support vector machine

Support vector machine (SVM) is a non-linear model, which have been used for the well-known emotion algorithms and support vector classifier separates the emotional states with a maximal margin. The advantage of support vector classifier is that it can be extended to non-linear boundaries by the kernel trick. SVM supervised learning models with associated learning algorithms that analyze data and recognize patterns, used for classification and regression analysis. SVM is designed for a two-class classification by finding the optimal hyperplane where the expected classification error of test samples is minimized and has been utilized as a pattern classifier to overcome the difficulty in pattern classification due to the large amount of within-class variation of features and the overlap between classes, although the features are carefully extracted [29]. The goal in training SVM is to find the separating hyperplane with the largest margin. We expect that the larger the margin, the better generalization of the recognizer [30].

SVM [26, 31] finds a hyperplane based on support vector to analyze data and recognize patterns. The complexity of the resulting classifier is characterized by the number of support vectors rather than the dimensionality of the transformed space. The goal in training SVM is to find the separating hyperplane with the largest margin. We expect that the larger the margin, the better the generalization of the classifier. The distance from any hyperplane to a pattern y is |g(y)|/||a||, and assuming that a positive margin b exists

8 $$ {z}_kg\left({\mathrm{y}}_k\right)/\left\Vert \mathrm{a}\right\Vert \ge \mathrm{b},\kern0.5em k=1,\kern0.5em \dots, \kern0.5em \mathrm{n}; $$

The goal is to find the weight vector a that maximizes b. Here, z_*k*_ is the class of *k*th pattern, b is the margin, and g(y) is a linear discriminant in an augmented y space,

9 $$ \mathrm{g}\left(\mathrm{y}\right)={\mathrm{a}}^{\mathrm{T}}\mathrm{y} $$

The parameters of the maximum-margin hyperplane are derived by solving the optimization. There exist several specialized algorithms for quickly solving the QP problem that arises from SVMs, mostly relying on heuristics for breaking the problem down into smaller pieces. The SVM algorithm has been widely applied in the biological and other sciences.

## Experimental result

For the results of psychological assessment, we analyzed the intensity of each emotion that the participants experienced. The average (SD) intensity of boredom was 5.23(1.35), pain 5.74(0.71), and surprise 6.35(0.69).

### Physiological responses induced by emotions

**Fig. 1:**
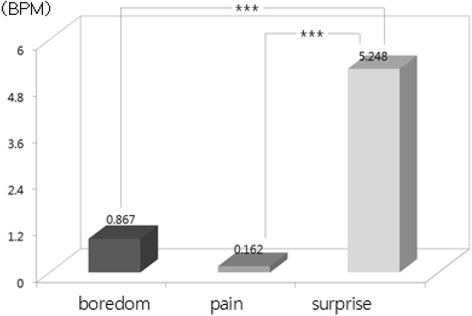
Difference among emotions in HR (***p < .001)

**Fig. 2:**
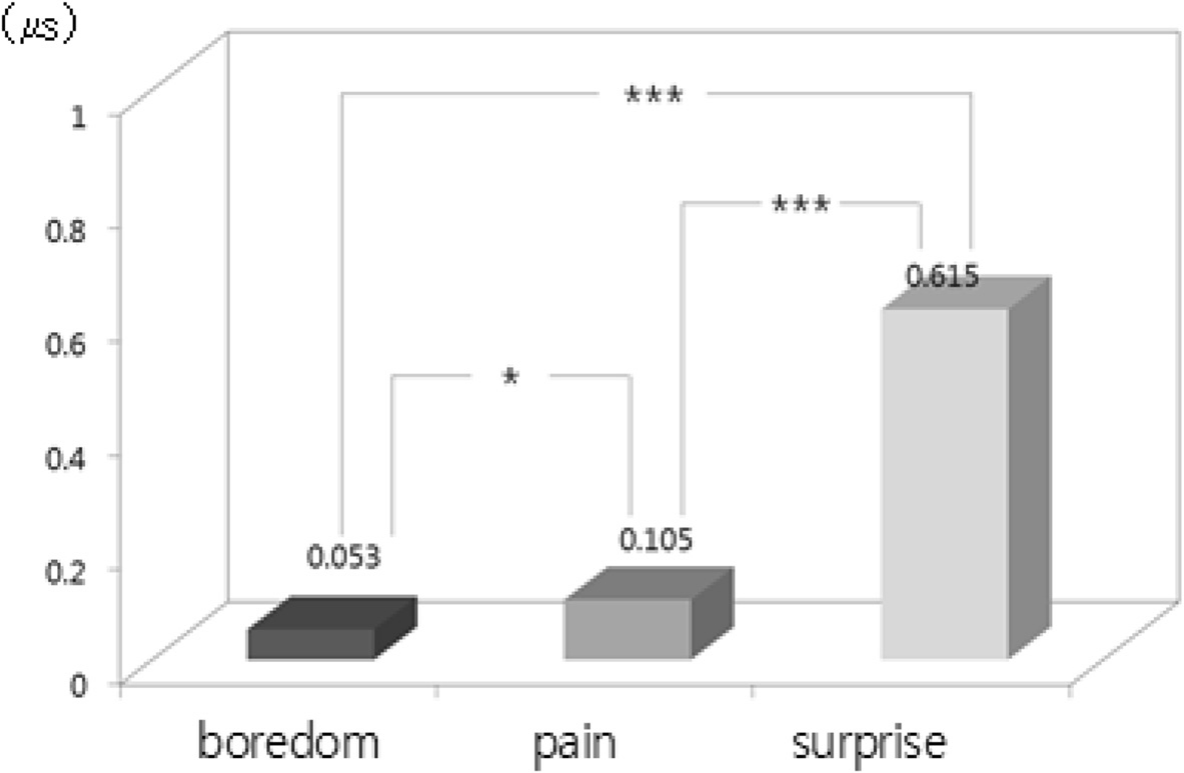
Difference among emotions in SCL (*p < .05, ***p < .001)

**Fig. 3:**
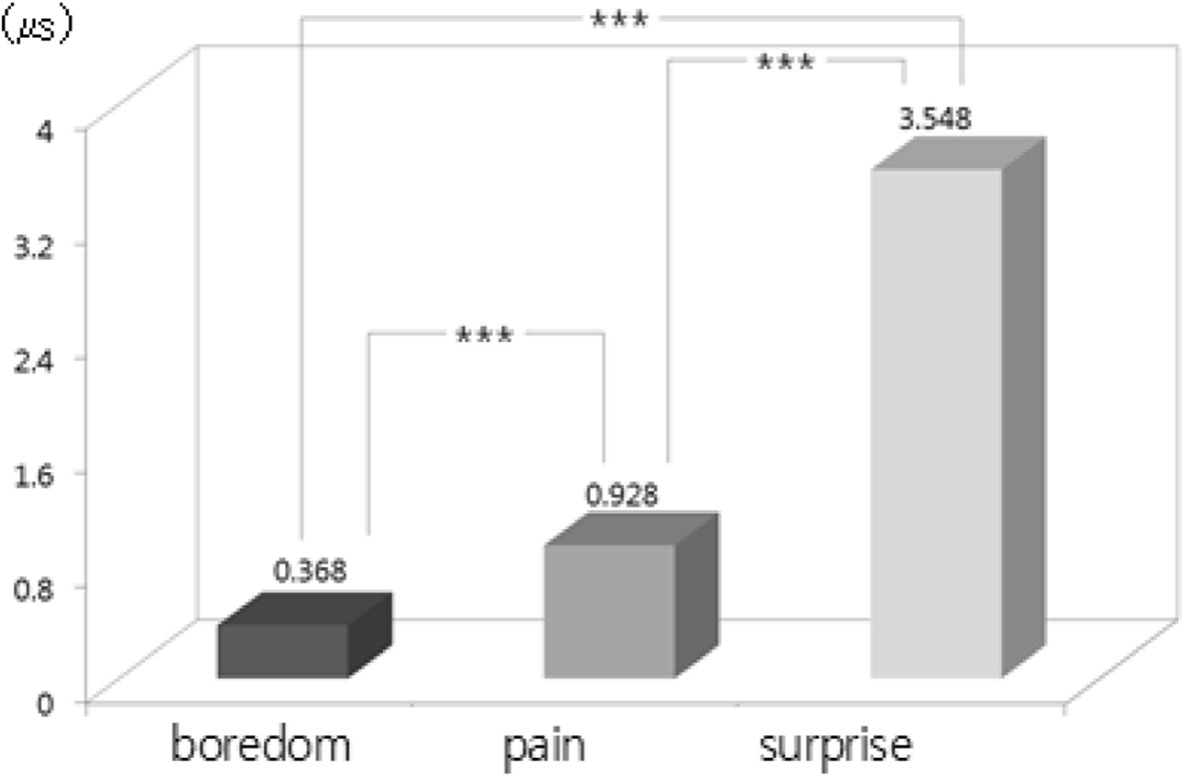
Difference among emotions in SCR (***p < .001)

**Fig. 4:**
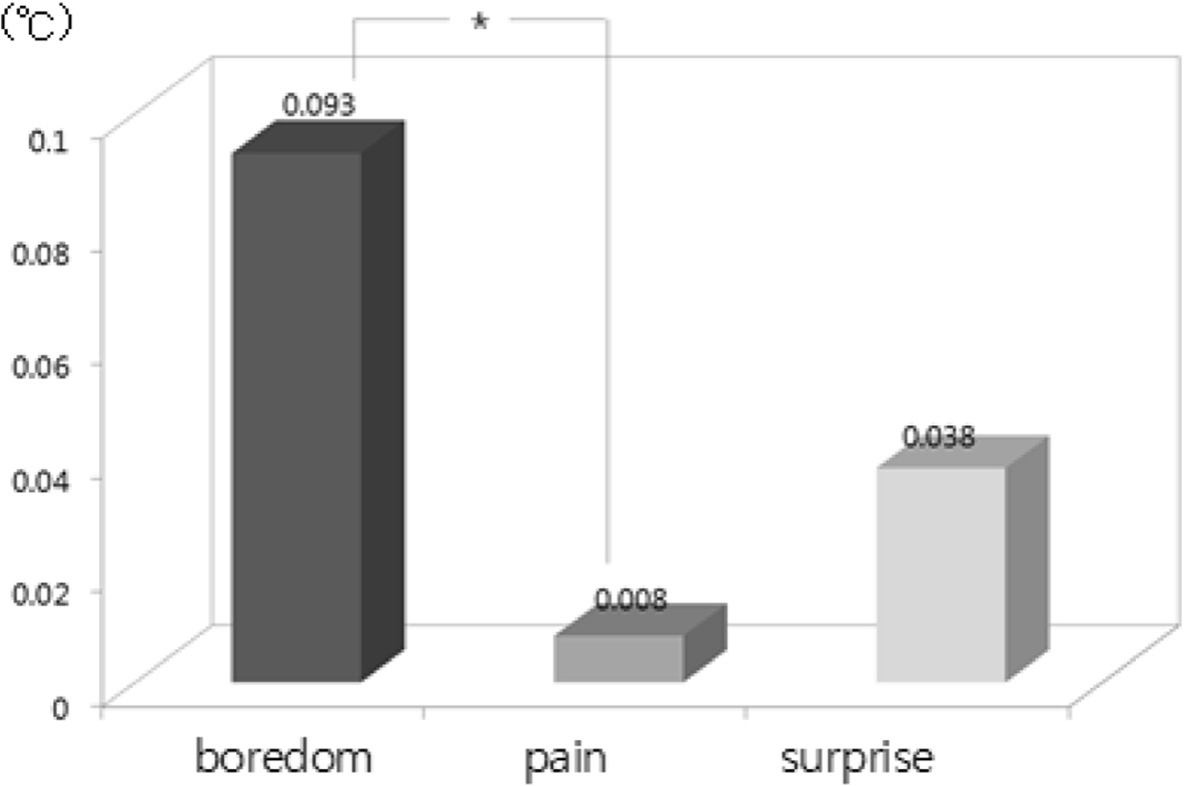
Difference among emotions in meanSKT (*p < .05)

**Fig. 5:**
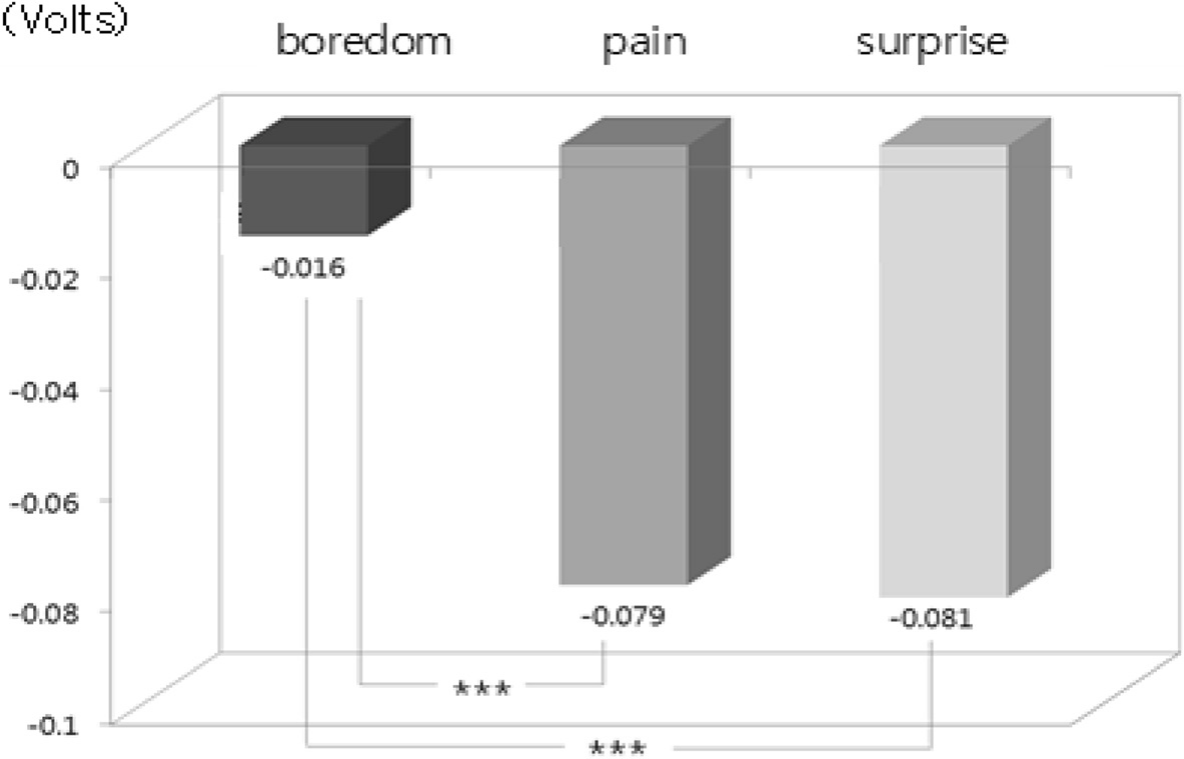
Difference among emotions in BVP (***p < .001)

**Fig. 6:**
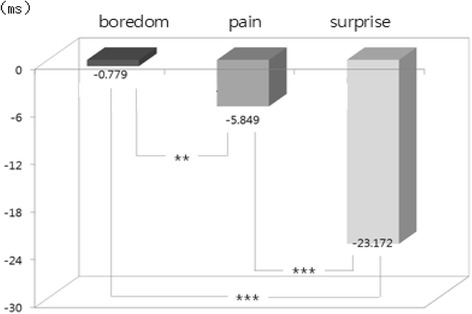
Difference among emotions in PTT (**p < .01, ***p < .001)

The one-way ANOVA and LSD post-hoc test (by SPSS 15.0) were used to examine difference among emotions from physiological signals. Result showed that there were statistically significant differences among emotions in HR [*F*(2, 534) = 31.37, *p* = .000], SCL [*F*(2, 534) = 294.10, *p* = .000], SCR [*F*(2, 534) = 277.54, *p* = .000], BVP [*F*(2, 534) = 48.60, *p* = .000], and PTT [*F*(2, 534) = 132.20, *p* = .000]. We used bar graphs to plot the comparison of the significant differences among emotions (i.e., results of LSD post-hoc test). The bars display the changes of emotional state from the baseline state (i.e., emotional state minus baseline state) in nine physiological features such as d_HR, d_SCL and d_SCR, etc. shown in Table 2. The upward bars show the means of physiological response during when emotional state was higher and downward bars show the means of physiological response during when emotional state was lower compared to baseline. In LSD post-hoc tests, the increased HR in surprise was significantly higher in boredom and pain (*p* < .001) (Fig. 1). Regarding the EDA response, SCL and SCR changes showed significant differences among emotions. SCL and SCR during surprise were significantly higher than boredom and pain (*p* < .001). In addition to that, SCL and SCR during pain were significantly higher than boredom (SCL *p* < .05; SCL *p* < .001) (Figs. 2 and 3). The change in meanSKT, during boredom was higher in pain (*p* < .05) and there was no significant difference between boredom and surprise or between pain and surprise (Fig. 4). On the other hand, the value of BVP signals during pain and surprise were significantly decreased than during boredom (*p* < .001) and PTT in surprise showed a significant decrease compared to during boredom and pain (*p* < .001) (Figs. 5 and 6).

### Results of emotion recognition

In the assessment of the performance of the five well-known recognizers, we used the recognition accuracy for emotions. We used the Classification Toolbox of MATLAB for CART and Naïve Bayes, and Duda’s Toolbox (http://www.yom-tov.info/computer_manual.html) for LDA and SVM. SOM toolbox (www.cis.hut.fi/projects/somtoolbox/) is available in MATLAB. We used feature normalization, and default values implemented in the toolbox were used for the related parameters of algorithms. The classification conducted in the study underwent a 10-fold cross-validation, which is a statistical method for evaluating models. In 10-fold cross-validation, the original sample is randomly partitioned into 10 equal size subsamples. Nine subsamples of 10 are used as training data, and 1 subsample is used as the testing data. Next, the process is repeated 10 times (the 10 folds), with each of the 10 subsamples are used exactly once as the testing data. Table 3 shows the recognition accuracy as a percentage (%) in algorithms. The results show that 84.7 % of test cases can be correctly classified by DFA as a statistical method. In the results of machine learning algorithms, the classification rates using LDA, CART, SOM, Naïve Bayes, and SVM were 74.9, 67.8, 61.5, 71.9, and 62.0 %, respectively. For emotion recognition, the statistical method showed more accurate recognition accuracy compared to the machine learning algorithms. As a result, DFA was the optimal method to classify three emotions, i.e., boredom, pain, and surprise.

**Table 3:** Results of emotion classification by all algorithms

Models	Accuracy (%)
DFA (SPSS 15.0)	84.7
LDA	74.9
CART	67.8
SOM	61.5
Naïve Bayes	71.9
SVM	62.0

*DFA* discriminant function analysis, *LDA* linear discriminant analysis, *CART* classification and regression trees, *SOM* self-organizing map, *SVM* support vector machine

Table 4 shows the classification results about DFA, i.e., 76.5 % in pain, 89.5 % in boredom, and 88.9 % in surprise. The LDA provided 74.9 % of accuracy in total and regarding the accuracy in each emotion, pain was recognized with 76.3 %, boredom 75.6 %, and surprise 72.9 % (Table 5). In analysis of CART, accuracy of each emotion had a range from 58.9 to 76.1 %, and classification accuracy of 69.2 % was achieved in pain, 76.1 % in boredom, and 58.9 % in surprise (Table 6). In Table 7, SOM showed recognition accuracy of 69.3, 52.7, and 62.0 in pain, boredom, and surprise, respectively. The result of the Naïve Bayes was 71.9 % in all emotions and this algorithm successfully recognized 77.8 % in pain, 71.6 % in boredom, and 66.7 % in surprise (Table 8). Finally, the result of SVM showed that classification accuracy of 67.0, 62.1, and 57.3 % according to the order of pain, boredom, and surprise (Table 9).

**Table 4:** Result of emotion classification by DFA

(%)	Pain	Boredom	Surprise
Pain	76.5	23.5	0.0
Boredom	7.9	89.5	2.6
Surprise	3.7	7.4	88.9

**Table 5:** Result of emotion classification by LDA

(%)	Pain	Boredom	Surprise
Pain	76.3	1.8	21.9
Boredom	5.7	75.6	18.8
Surprise	20.8	6.3	72.9

**Table 6:** Result of emotion classification by CART

(%)	Pain	Boredom	Surprise
Pain	69.2	6.5	24.3
Boredom	6.8	76.1	17.0
Surprise	21.9	19.3	58.9

**Table 7:** Result of emotion classification by SOM

(%)	Pain	Boredom	Surprise
Pain	69.3	10.2	20.5
Boredom	3.0	52.7	44.4
Surprise	7.8	30.2	62.0

**Table 8:** Result of emotion classification by Naïve Bayes

(%)	Pain	Boredom	Surprise
Pain	77.8	2.3	19.3
Boredom	8.3	71.6	19.5
Surprise	16.1	16.7	66.7

**Table 9:** Result of emotion classification by SVM

(%)	Pain	Boredom	Surprise
Pain	67.0	8.0	25.0
Boredom	5.9	62.1	32.0
Surprise	14.1	28.6	57.3

## Discussion

We identified the specific responses of different emotions, i.e., boredom, pain, and surprise, based on the physiological signals. These emotions were then classified using a statistical method and five emotion recognizers.

### Physiological responses induced by emotions

Our purpose was to identify the differences of physiological responses among boredom, pain, and surprise. We have measured physiological signals while inducing different emotions by emotional stimuli and have statistically verified that HR, SCL, SCR, meanSKT, BVP, and PTT among physiological signals are meaningful features being able to identify the differences of these emotions. In analysis based on this features, emotion-specific responses induced by each emotional stimulus are as follows.

In boredom, there was a significant increase of meanSKT. Skin temperature serves as a surrogate marker of blood flow changes that result from vascular reactivity. SKT is influenced mainly by sympathetic adrenergic vasoconstrictor nerves, and increased SKT in our result means vasodilation through withdrawal of neural activity. For example, the more tense muscles under strain, the blood vessels would be contracted and the temperature would decrease. Increased SKT in boredom is supported by previous studies [32–34]. Skin temperature shows an extreme decrease by stress and fear and increase during relaxation, boredom, and sleep. In particular, skin temperature shows a great change under emotional stress (i.e., rapid decrease and rapid return) [32–34]. We observed that pain and surprise were associated with mild increased SKT instead of extreme changes. This might be caused by our period for the data analysis, i.e., a 30-s emotional state. SKT is considered a relatively slow indicator of changes in emotional state. We might have a longer time for emotional state in order to confirm overall changes in SKT as in previous studies, which make us obtain extreme changes of SKT.

Physiological responses induced by pain showed a greatly decreased BVP, mildly decreased PTT, and mildly increased both SCL and SCR. The BVP is the measure of the amount of blood currently running though the vessels in the finger of participants, and provides an activity on vasoconstriction. Changes in the BVP signal could indicate relative changes in the vascular bed due to vasodilation or vasoconstriction (increase or decrease in blood perfusion) as well as changes in the elasticity of the vascular walls, reflecting changes in blood pressure [35]. Decrease in BVP amplitude during pain compared to the baseline state might be implying peripheral vasoconstriction in the finger associated with arousal [36, 37]. Regarding the PTT, it is the measure of the elapsed time between the R-wave of the ECG and the arrival of the pulse wave at the finger [38]. It is affected by changes in the contractile force of the heart and changes in the mean arterial blood pressure. Since the increased PTT is linked to a suppression of sympathetic activation, the great decrease of PTT during pain in the study could reflect the sympathetic activation. Additionally, increased SCL and SCR mean that the skin is sweaty and the sympathetic nervous system is activated. In particular, SCL and SCR are related to sympathetic-adrenal-medullary (SAM) activation which indicates the pain progress. In conclusion, we confirmed that pain-specific responses were caused by the SAM activation and peripheral vasoconstriction. These findings are associated with physical pain since this study was focused on physical pain instead of psychological pain as described in the introductory section.

Surprise-specific response significantly increased SCL, SCR, and HR as well as greatly decreased BVP and PTT. In a review article, Kreibig [13] has reported that surprise is associated with short-term duration SCR of medium response size and characterized by rapid increase and rapid return of SCR [22], increased SCL [39], increased HR [38–41], decreased [41] or increased finger temperature [22, 39]. Increased SCL and SCR have been proposed to reflect cognitively or emotionally mediated motor preparation [42] and an increase in action tendency [4, 43]. HR is controlled by the central nervous system, which varies the impulse traffic in the sympathetic and parasympathetic nerve fibers terminating in the SA node. HR is the result of the intrinsic automaticity of the SA node and the modulating influence of the autonomic nervous system [44, 45]. During relatively mild work, HR effects primarily by a withdrawal of parasympathetic restraint on the SA node. At higher levels of work, further withdrawal of parasympathetic restraint occurs, but increases in sympathetic activity become progressively more important in accelerating the cardiac rate. In other words, increased HR means a sympathetic activation (related to fight/flight) to prepare for action and decreased HR the parasympathetic activation (related to relaxation) as signals for resting and recovery. In our results, there were great decreases of BVP and PTT during surprise. These responses derived from vasoconstriction are considered as by α-adrenergic stimulus [46]. As noted above, decreased BVP reflects peripheral vasoconstriction in the finger and great decrease of PTT implies strong sympathetic activation. In sum, physiological responses during surprise could be characterized by strong sweat activity, vasoconstriction, and increased heart rate activated by the sympathetic nervous system.

### Results of emotion recognition

Our study demonstrated the possibility of emotion recognition for boredom, pain, and surprise based on physiological signals. To examine the optimal method to effectively classify these emotions, we used both a statistical method and machine learning algorithms. The results showed that the classification rate by DFA was 84.7 %. The DFA was the best recognition method to classify the three emotions. In the result of machine learning algorithms, emotion recognition by LDA was the highest as 74.9 %. As mentioned earlier in the “Emotion recognition methods” section, DFA was the statistical method composed of a discriminant function based on linear combinations of independent variables. Those independent variables provide the best discrimination between classification groups. The LDA algorithm also looks for linear combinations of variables which best explain the way of data classification. Linear methods find the vectors in the underlying space that best discriminate among classes and tries to maximize the between-class differences and minimize the within-class ones. Also, they are good at discriminating different classes because it is a surveillance method. As a result, the emotions appear to be accurately classified by linear methods compared to non-linear methods such as SOM, CART, etc. Linear methods offer many advantages in other pattern recognition such as face or speech recognitions. On the other hand, classification rates by other algorithms had a range from 61.5 to 71.9 %. It is assumed that the overlapped tendency in non-linear methods could influence the low classification rate.

## Conclusion

In conclusion, we examined the difference of three emotions, i.e., boredom, pain, and surprise, based on physiological signals and the possibility for the classification of the three emotions by using six classification methods. This study has a few limitations. Firstly, the physiological and classification results on pain or surprise are limited to the overall generalization since the physical pain was focused as excluding psychological pain and startle reflex as surprise reaction not the surprise response that could be caused by any psychological processes in the study. As we described in the introduction, psychological pain or surprise are relatively complex (e.g., psychological pain is likely to be a mixture of sadness and fear) making it hard to observe the emotion-specific physiological responses. In the further study, it might be required to divide pain or surprise emotions in details as physical or psychological pain and pleasant or unpleasant surprise. Secondly, the recognition accuracy of machine learning algorithms used in the study was relatively lower, particularly in the non-linear methods such as SOM and CART, than the linear methods. We confirmed that for the classification of the three emotions, the algorithms based on the linear method was more effective rather than based on the non-linear method. To improve the classification accuracy of emotions, it might be needed to extract new physiological features (e.g., changes of SKT over time) and to modify the testing method in order to discriminate the three emotions accurately.

Despite a few limitations, the findings in the study could contribute to expanding the understanding of human affective states and the underlying physiological mechanism. The results help us develop and expand emotion models on emotion-specific physiological patterns by adding the emotion-specific physiological responses for non-basic emotion in addition to the basic emotion observed by previous researchers. In the further study, if the gender or age effects in the physiological responses of boredom, pain, and surprise are included, the findings could contribute to anthropology by revealing humans’ emotional response characteristics by different groups. The recognition of various emotions as well as different groups could be applied to develop user-friendly emotional interaction system between human and computer or machine in affective computing and HCI.
